# Supplementary material for: Positive Affect Moderates the Influence of Perceived Stress on the Mental Health of Healthcare Workers during the COVID-19 Pandemic
Source: Int J Environ Res Public Health. 2022 Oct 20;19(20):13600. doi: 10.3390/ijerph192013600 (PMC9603543; doi:10.3390/ijerph192013600)
Supplement: Supplementary file 1 [file ijerph-19-13600-s001.zip › ijerph-1978838-supplementary.pdf]

Table S1. Moderating effect of positive affect on the relationship between stress and depression in the low-stress group.

	Model1		Model2		Model3		Model4	
	b	***p***	***b***	***p***	***b***	***p***	***b***	***p***
Gender (Female)	0.031	0.950	-0.205	0.646	-0.197	0.660	-0.197	0.661
Age	-0.105	0.180	-0.068	0.324	-0.067	0.334	-0.066	0.346
Education level (Junior high or below)								
High school and vocational school	3.634	0.161	3.375	0.142	3.356	0.144	3.417	0.148
College or above	4.272	0.093	4.152	0.066	4.185	0.064	4.249	0.068
Marital status (Single)								
Married	0.374	0.573	0.425	0.437	0.466	0.398	0.465	0.399
Divorced or other	1.549	0.363	1.883	0.212	1.917	0.205	1.938	0.204
Status of residence (Not living alone)	-0.071	0.912	0.044	0.939	0.061	0.915	0.064	0.911
Income (yuan, < 5,000)								
5,000–10,000	-0.358	0.589	-0.434	0.459	-0.408	0.488	-0.415	0.484
> 10,000	0.149	0.838	-0.239	0.712	-0.198	0.761	-0.209	0.752
Professional title (Junior title)								
Intermediate title	-0.961	0.122	-0.771	0.161	-0.776	0.159	-0.781	0.159
Senior title	1.575	0.015*	1.416	0.014*	1.429	0.013*	1.420	0.014*
Seniority	-0.056	0.380	-0.090	0.112	-0.093	0.104	-0.093	0.104
**Stress (A)**			**0.491**	**<0.001*****	**0.473**	**<0.001*****	**0.480**	**<0.001*****

	Model1		Model2		Model3		Model4	
	b	*p*	*b*	*p*	*b*	*p*	*b*	*p*
**Positive affect (B)**					**-0.026**	**0.516**	**-0.037**	**0.719**
**A × B**							**-0.002**	**0.906**
**Model *F* (*p*)**	**2.962(0.001)**		**8.179(<0.001)**		**7.605(<0.001)**		**7.066(<0.001)**	
R^2 (ΔR^2)	**0.141**		**0.331(0.190)**		**0.332(0.001)**		**0.332(0.000)**	
ΔF (*p*)			**60.930(<0.001)**		**0.423(0.516)**		**0.014(0.906)**	

*: $p < 0.05$; **: $p < 0.01$; ***: $p < 0.001$

The variance inflation factor (VIF) of Model4 was 1.02-3.35 (< 10).

Model1: the regression with sociodemographic variables.

Model2: the regression with perceived stress after controlling the sociodemographic variables.

Model3: the regression with perceived stress and positive affect after controlling the sociodemographic variables.

Model4: the regression with interaction term of stress × positive affect after controlling the sociodemographic variables.

Table S2. Moderating effect of positive affect on the relationship between stress and anxiety in the high-stress group.

	**Model1**		**Model2**		**Model3**		**Model4**	
	b	***p***	***b***	***p***	***b***	***p***	***b***	***p***
Gender (Female)	0.390	0.363	0.400	0.272	0.304	0.402	0.305	0.402
Age	0.029	0.562	-0.002	.966	-0.016	0.701	-0.018	0.676
Education level (High school and vocational school)								
College or above	0.684	0.224	0.568	0.234	0.516	0.276	0.560	0.243
Marital status (Single)								
Married	0.454	0.407	0.596	0.199	0.664	0.151	0.700	0.133
Divorced or other	2.018	0.297	1.666	0.310	1.700	0.298	1.741	0.287
Status of residence (Not living alone)	-0.790	0.120	-1.018	0.018*	-0.998	0.020*	-1.014	0.019*
Income (yuan, < 5,000)								
5,000–10,000	-0.448	0.385	-0.133	0.762	-0.069	0.874	-0.095	0.829
> 10,000	0.371	0.554	0.590	0.267	0.645	0.223	0.619	0.244
Professional title (Junior title)								
Intermediate title	0.919	0.065	0.175	0.681	0.207	0.626	0.189	0.656
Senior title	0.739	0.158	0.377	0.395	0.383	0.386	0.378	0.392
Seniority	-0.026	0.514	0.016	0.641	0.023	0.498	0.024	0.482
**Stress (A)**			**0.499**	**<0.001*****	**0.477**	**<0.001*****	**0.465**	**<0.001*****
**Positive affect (B)**					**-0.075**	**0.015***	**-0.058**	**0.159**
**A × B**							**-0.004**	**0.525**
**Model *F* (*p*)**	**1.637(0.086)**		**15.940(<0.001)**		**15.350(<0.001)**		**14.260(<0.001)**	

	Model1		Model2		Model3		Model4	
	b	***p***	***b***	***p***	***b***	***p***	***b***	***p***
R^2 (ΔR^2)	**0.041**		**0.313(0.272)**		**0.323(0.010)**		**0.323(0.000)**	
ΔF (p)			**166.18(<0.001)**		**5.984(0.015)**		**0.406(0.525)**	

*: $p < 0.05$; **: $p < 0.01$; ***: $p < 0.001$

The variance inflation factor (VIF) of Model4 was 1.03-1.81 (< 10).

Model1: the regression with sociodemographic variables.

Model2: the regression with perceived stress after controlling the sociodemographic variables.

Model3: the regression with perceived stress and positive affect after controlling the sociodemographic variables.

Model4: the regression with interaction term of stress × positive affect after controlling the sociodemographic variables.

Table S3. Moderating effect of positive affect on the relationship between stress and anxiety in the low-stress group.

	**Model1**		**Model2**		**Model3**		**Model4**	
	b	***p***	***b***	***p***	***b***	***p***	***b***	***p***
Gender (Female)	0.061	0.897	-0.185	0.645	-0.183	0.651	-0.183	0.651
Age	-0.019	0.797	0.019	0.758	0.020	0.754	0.016	0.805
Education level (Junior high or below)								
High school and vocational school	2.916	0.227	2.647	0.199	2.642	0.201	2.393	0.259
College or above	3.323	0.160	3.199	0.114	3.209	0.113	2.944	0.159
Marital status (Single)								
Married	0.240	0.675	0.320	0.513	0.333	0.501	0.335	0.500
Divorced or other	1.167	0.461	1.514	0.264	1.524	0.262	1.441	0.293
Status of residence (Not living alone)	-0.081	0.893	0.039	0.940	0.044	0.932	0.032	0.950
Income (yuan, < 5,000)								
5,000–10,000	0.252	0.682	0.174	0.742	0.181	0.731	0.211	0.692
> 10,000	0.789	0.246	0.387	0.506	0.399	0.500	0.443	0.455
Professional title (Junior title)								
Intermediate title	-1.013	0.080	-0.816	0.099	-0.817	0.100	-0.796	0.110
Senior title	0.933	0.120	0.769	0.134	0.772	0.133	0.806	0.121
Seniority	-0.090	0.132	-0.125	0.015*	-0.126	0.015*	-0.124	0.016*
**Stress (A)**			**0.509**	**<0.001*****	**0.504**	**<0.001*****	**0.476**	**<0.001*****

	Model1		Model2		Model3		Model4	
	b	*p*	*b*	*p*	*b*	*p*	*b*	*p*
**Positive affect (B)**					**-0.008**	**0.829**	**0.038**	**0.681**
**A × B**							**0.006**	**0.591**
**Model *F* (*p*)**	2.067(0.020)		**8.865(<0.001)**		**8.199(<0.001)**		**7.646(<0.001)**	
R^2 (ΔR^2)	0.103		**0.349(0.247)**		**0.349(0.000)**		**0.350(0.001)**	
ΔF (*p*)			**81.234(<0.001)**		**0.047(0.829)**		**0.289(0.591)**	

*: $p < 0.05$; **: $p < 0.01$; ***: $p < 0.001$

The variance inflation factor (VIF) of Model4 was 1.02-3.35 (< 10).

Model1: the regression with sociodemographic variables.

Model2: the regression with perceived stress after controlling the sociodemographic variables.

Model3: the regression with perceived stress and positive affect after controlling the sociodemographic variables.

Model4: the regression with interaction term of stress × positive affect after controlling the sociodemographic variables.

Table S4. Items and instruction for Perceived Stress Scale (PSS-10).

Items	Never	Almost never	Sometimes	Fairly often	Very often
1. In the last month, how often have you been upset because of something that happened unexpectedly?	0	1	2	3	4
2. In the last month, how often have you felt that you were unable to control the important things in your life?	0	1	2	3	4
3. In the last month, how often have you felt nervous and 'stressed'?	0	1	2	3	4
4. In the last month, how often have you felt confident about your ability to handle your personal problems?	0	1	2	3	4
5. In the last month, how often have you felt that things were going your way?	0	1	2	3	4
6. In the last month, how often have you found that you could not cope with all the things that you had to do?	0	1	2	3	4
7. In the last month, how often have you been able to control irritations in your life?	0	1	2	3	4
8. In the last month, how often have you been able to control irritations in your life?	0	1	2	3	4
9. In the last month, how often have you been angered because of things that happened that were outside of your control?	0	1	2	3	4
10. In the last month, how often have you felt difficulties were piling up so high that you could not overcome them?	0	1	2	3	4

Table S5. Items and instruction for Generalized Anxiety Disorder 7-item Scale (GAD-7).

Over the last 2 weeks, how often have you been bothered by the following problems?	Not at all	Several days	More than half the days	Nearly every day
1. Feeling nervous, anxious or on edge.	0	1	2	3
2. Not being able to stop or control worrying.	0	1	2	3
3. Worrying too much about different things.	0	1	2	3
4. Trouble relaxing.	0	1	2	3
5. Being so restless that it is hard to sit still.	0	1	2	3
6. Becoming easily annoyed or irritable.	0	1	2	3
7. Feeling afraid as if something awful might happen.	0	1	2	3

Table S6. Items and instruction for Patient Health Questionnaire-9 (PHQ-9).

Over the last 2 weeks, how often have you been bothered by any of the following problems?	Not at all	Several days	More than half the days	Nearly every day
1. Little interest or pleasure in doing things.	0	1	2	3
2. Feeling down, depressed, or hopeless.	0	1	2	3
3. Trouble falling or staying asleep, or sleeping too.	0	1	2	3
4. Feeling tired or having little energy.	0	1	2	3
5. Poor appetite or overeating.	0	1	2	3
6. Feeling bad about yourself — or that you are a failure or have let yourself or your family down	0	1	2	3
7. Trouble concentrating on things, such as reading the newspaper or watching television.	0	1	2	3
8. Moving or speaking so slowly that other people could have noticed? Or the opposite — being so fidgety or restless that you have been moving around a lot more than usual.	0	1	2	3
9. Thoughts that you would be better off dead or of hurting yourself in some way.	0	1	2	3
